# Remarks on the Unity of the Body, as Illustrated by Some of the More Striking Phenomena of Sympathy, &c.

**Published:** 1836-04-01

**Authors:** 


I. Remarks on the Unity of the Body, as illustrated by some of the more striking Phenomena of Sympathy, both mental and corporeal; with a view of enlarging the Grounds, and improving the Application of the Constitutional Treatment of Local Diseases. By *George Macilwain*, M.R.C.S. Surgeon to the Finsbury Dispensary. Octavo, pp. 294. Highley, 1836.

II. (Retrospective) Practical Observations on the Action of Morbid Sympathies, as included in the Pathology of certain Diseases. By *Andrew Wilson*, M.D. (of Kelso.) Octavo, pp. 407. London, 1818.

The first work on our list is a course of lectures on sympathy—a subject of great importance to the surgeon and the physician—if such an absurd division be still judged necessary. In this country, the doctrine of sympathy has engaged the attention, particularly, of Whytt, Monro, Wilson (of Kelso), Alison, Abernethy, Charles Bell—and lastly, our present author. Haller, on the Continent, was the most distinguished writer on this intricate subject; and, in his time, many an abstruse theory was engendered and broached to explain the cause of this mysterious consent between distant as well as contiguous parts. There are some—perhaps many, who still deny that anatomy—that is to say, the communication of nerves—can explain the cause or the laws of sympathy. Even the ingenious and important discovery of Sir C. Bell will not satisfactorily account for the sympathies between distant parts—say the stomach and kidneys—by direct nervous communication. But still, when we reflect that impressions on every part of the body may, or rather *must* be transmitted to the brain—the common sensory—we cannot well come to any other conclusion than that the nervous system is, after all, the medium of communication, both in health and disease—even where no direct intercommunication of nerves can be traced by the anatomist.

A review of the very instructive work on "morbid sympathies," by Dr. Wilson, of Kelso, appeared in an early series of this Journal, (January, 1819,) which had then but a comparatively limited circulation, and is probably unknown to nine-tenths of our present readers. The original work had a still more limited circulation, and may be said to have fallen "still-born" from the press! Yet it is a valuable production, and we are induced to re-publish a brief analysis of it in this place. In the 6th volume of our analytical series (January 1827). will be found a full account of Dr. Alison's paper on this subject, published in the second volume of the Edinburgh Transactions of the Medico-chirurgical Society. To this we may refer our present readers. The review was drawn up by a highly talented physician, now, alas, no more! On reperusing it, we find some sentiments, with which we cannot entirely concur, but still we can point to it with pleasure, as the production of a lost and lamented contributor.

Mr. Macilwain is a disciple and admirer of Abernethy—and has imbibed many opinions of that talented but half-mad surgeon. From the following passage it would appear that Mr. Macilwain does not attach any very great degree of importance to ANATOMY, as the means of elucidating sympathy.

" It cannot, perhaps, de denied, that anatomy has furnished some assistance in enquiring into the phenomena of Sympathy ; but it is to be feared that more has been expected from it than it is capable of supplying ; whilst, by engrossing too exclusive an attention, it may sometimes have closed more enlarged, and perhaps more promising, avenues for investigation. In pointing out universal nervous communications, it suggests, a priori, the functional integrity of the body ; in shewing identical tissues, arranged in different situations, and, in assisting our physiological conclusions as to individual organs, it facilitates the classification of phenomena ; applied to the explanation of individual sympathies, it affords us little help : if it occasionally hold out promise in this way, it is but to allure us to disappointment.

The consideration of the eighth pair of nerves and its communications with the branches of the cervical ganglia of the sympathetic, and the sympathies of certain parts to which these are distributed ; and the inadequacy of any application, I may say the absence of analogous facts, when we consider other sympathies no less remarkable, the uterus and stomach, for example,—illustrate this remark.

We do not indeed expect anatomy to assist us in explaining the most marked sympathy of all ; namely, that between mind and body ; but its failure here is but typical of its incompetency to explain many *corporeal* phenomena. We shall probably obtain more advantage from a careful observation and notation of the phenomena which are daily unfolded to us ; of the circumstances by which they are preceded, accompanied, or followed ; and, when our facts shall have become multiplied, by examining how far we can establish that co-existence or sequence of any of them, which shall suggest the idea of a law, in the mode of their production." 11.

This is nearly the opinion of Dr. Wilson, as will be seen in the sequel ; but still there can be little doubt that the nerves are the medium of communication, the brain itself being the vinculum.

Sympathies are natural or morbid. The *latter* are, no doubt, only perversions or modifications of the *former*. They are *reciprocal*, as between the head and stomach—stomach and bowels—skin and kidney. At other times they seem to act chiefly in one direction. The testicle will sympathise with the urethra, and the stomach with the testicle—but rarely in the opposite direction. Sympathetic affections are sometimes more prominent than the primary impressions—as when the head sympathises with the stomach or liver—the knee with the hip—or the shoulders with the liver. At other times the secondary effect is less strong than the original.

Our author divides sympathies into general and particular. The *former* is illustrated by " constitutional irritation," in which the whole system sympathises with all its parts. " This, indeed, is the "*unity* of the body," which forms the chief feature of the title-page. The specific sympathies he divides according as they appear to be more or less influenced by function, structure, and other known or unknown circumstances.

As the work is in the form of lectures, and has been delivered to pupils, it contains a great deal of minute but common-place observations, which we cannot make use of in a review. There is no doubt, however, that the matter was well adapted to the audience, and very useful too.

The MUCOUS MEMBRANES present a chain of sympathies as well marked as any in the body. The different parts not only sympathise with each other, but they sympathise with the skin in a very remarkable manner. Our author thinks, and probably with justice, that a great majority of cutaneous diseases are dependent on this sympathy. The following case is related in illustration of the sympathy existing among the fibrous structures.

"A poor boy, not very well clad, was sitting on a gate on a cold day, and he felt the wind blow on his thigh, which was quickly followed by a very acute pain in the same situation. He immediately went home, which he reached with difficulty; and on the second day his parents applied at the Dispensary. On examining his thigh, it was neither red nor swollen, or very little, but extremely painful, and exquisitely tender on the slightest pressure. I told the gentlemen who accompanied me that I regarded it as inflammation of the fascia. His health was much disordered, and his pulse indicated excitement with want of power. Leeches were freely applied to the part, with fomentations and poultices; and he was seen again the next day. The limb was now swollen, but not discoloured, excessively painful, and exhibited an appearance which led me to remark, that had it been in a woman recently parturient, it would be called phlegmasia dolens. The treatment consisted of further local depletion and mercury; but the rapidly increasing depression of the system, which had been observed from the first, obliged us to relinquish the plan, and endeavour to support him by such means as seem likely not to produce excitement. Notwithstanding the most sedulous attention to the conflicting indications, he gradually sank. Examination discovered a very considerable thickening of the fascia of the thigh, and of the cellular tissue: a moderate quantity of pus lay around and in the course of the femoral vein, which, on being laid open, was found also to contain pus, with considerable depositions of coagulable lymph." 22.

Tetanus and the spasm of rheumatism are adduced as exemplifying *muscular* sympathy. Voluntary muscles, however, are much less disposed to sympathise than the involuntary. The *serous* membranes, also, evince much less of this consent, than the *mucous*. Affections, however, of the former spread with great rapidity, and preserve a striking similarity. This is seen in peritoneal inflammation, &c.

Sympathy of the cellular tissues seems to be in common with the part to whose structure it contributes, or in whose vicinity it occurs. Its sympathies, however, are more salutary than morbid—and may be referred to one principle—the limitation of disease.

In the functional division our author refers to the sympathies of different parts of the alimentary canal, as shewn, for instance, in disordered stomach from disease in the rectum. As to the skin and mucous membranes, the two largest absorbing surfaces, in the body, "their sympathies are familiar, constant, and reciprocal." Over the cutaneo-pulmonic, and cutaneo-renal sympathies we pass. Mr. M. dwells on the sympathy between the skin and the heart; but we think rather mechanically—if not unsuccessfully. The same may be said of his cardiaco-renal sympathy. That between the heart and lungs is, in a great measure, mechanical—as indeed our author acknowledges.

The fourth chapter is on "intermediate sympathy"—or sympathy not to be connected with either structure or function. We do not find in this chapter much to extract, or perhaps to approve. Mr. M. falls into the error which is daily immolating thousands in these isles—the idea that cough and even fatal phthisis are often occasioned by stomach and liver disorder. It is difficult, of course, to ascertain the original *causes* of disorders; but we have, every day, reason to deplore the delusion of attributing cough and dyspnœa, and palpitation, nay, even copious purulent expectoration, to affections of the stomach and liver—thus overlooking the real, as well as obvious seat of the disease in the lungs! This oversight is now generally confined to those who despise a physical or stethoscopic examination of the chest. What experienced pathological observer will concur with our author in the following sentiment?

"It is *not often* we see the lungs affected without more or less derangement of the liver; nor the liver materially disordered without oppression of the respiratory organs. Their mechanical relations exert considerable influence in exciting a mutual sympathetic recognition of disturbance." 70.

We fearlessly assert that the liver is not affected in one case out of 100 of pulmonic disease.

The gastro-renal sympathy, Mr. M. thinks, takes place through the medium of the skin. The gastro-cardiac sympathy, he conceives to be produced through the medium of the brain. We apprehend that this sympathy is more direct than is imagined. We have watched it narrowly—and painfully. Flatus in the stomach will frequently produce intermission of the pulse, where the mind is intent on abstruse studies, and the intermission will instantly cease when the flatus has escaped.

In the sixth chapter, "on sympathy considered in its application to the history and treatment of disease," there are many judicious remarks, but not such as can be considered necessary to practitioners. They were very appropriate in a lecture-room. The chapter is interspersed with many practical cases of interest, from the Finsbury Dispensary. On mercurial abuses and diseases, our author is at home. The work winds up with an elaborate discourse vindicating his revered master, Mr. Abernethy, from the charge of materialism—or rather of confounding life with electricity. It is hardly necessary, at this time, to rip up these old matters. Let them rest quietly in the grave, or in oblivion. We may just quote one passage, however, which bears not very remotely on a subject which is now under discussion—the *natural* treatment of *fractures* without splints.

"Amongst the various misconstructions of Mr. Abernethy, it has been thought, that he disregarded local remedies; whereas he was fully acquainted with their just value. His treatment of susceptible surfaces, for example,—his mode of endeavouring to quiet local irritation, whilst he prosecuted his endeavours to tranquillize general disturbance, is another example of his attention to local matters, as it is of good surgery. In connexion with this, I may mention that no one could be more particular than he was, in keeping diseased joints motionless, by splints: a practice of great importance, and recently recommended by Sir Benjamin Brodie; but apparently without being aware of the fact I have just stated—as Mr. Abernethy is not mentioned." 272.

Now a fracture is a kind of diseased joint, *pro tempore*, and requires, in our humble apprehension, to be secured from motion, just as much as a knee with ulcerated cartilages—Mr. Radley to the contrary, notwithstanding. There is an ingenious tabular arrangement—or attempt at such—of the various sympathies;—but we fear that much additional observation is necessary before any tabular form can be depended on. Nevertheless we believe it to be as perfect as the present state of our knowledge will sanction. We recommend this work to junior practitioners, rather than to students; for, as things go, the latter have quite enough on their hands, without entangling themselves in the inextricable web of sympathies. By the way, our author might have introduced a new species of sympathy, peculiar to students—the vinculum being situated near Blackfriars!

---

In various parts of this Journal, but particularly in our review of Dr. Parry's Elements of Pathology, we took occasion to protest against the rage for tracing diseases almost exclusively to *vascular* derangement, and overlooking the morbid movements or state of the *nervous* system. The longer we live, the more we see, and the deeper we study, so much the more are we convinced, that not only are the *primary impressions* of morbific causes sustained by the *sentient* system of the human fabric, but that it is in *this* system the primary morbid *movements* first begin, and are thence propagated to the *vascular* system; which, from that moment, re-acts upon, and is again influenced by, the nervous system. We never shall have a sound pathology or successful practice, while any one of these two systems is studied in preference to the other; or, while they are considered as unlinked in perpetual reciprocity of action. Under this conviction, we shall give every possible publicity to the labours of those observers of Nature, who are exploring this interesting subject; and we could not have a better opportunity than in the analysis of Dr. Wilson's volume—a work which is characterized by deep thought, solid judgment, and accurate observation.

Dr. W. justly observes, that the laws of morbid sympathies are often very demonstrable to the senses, "and may be reasoned on in diagnostics with the utmost propriety, and considered as steady principles to be acted upon."

"We know, for instance, that certain irritating matter contained in the stomach, will very commonly produce a headache; but knowing also the universal influence which the stomach exercises over the whole human frame, if a person becomes affected with pain, &c. in a distant part of the body, in a limb, for instance, without any occasional cause having been applied, attended at the same time with certain signs of a loaded stomach, reasoning from the above facts, we readily believe, that if irritation in the stomach can produce pain in the forehead, it may, at any other time, produce pain in any other distant part also. It is a fair enough inference, therefore, that, in this case likewise, the primary disease is seated in the stomach, and is a cause of pain in the limb by an act of morbid sympathy." 8.

The brain and its appendages, indeed, being the medium through which the living principle acts upon the otherwise inert organic structure, as also the medium through which the mind is made conscious of injurious applications offered to any part of the machine, it may properly be said that every part of the human body is capable of sympathising with the rest; because we see that no part of it can admit of having the sense of feeling excited to the height of pain, without the whole frame being deranged in sensibility.* There, are, however, specific sympathies, which are so common in their oc-

---

* This sentiment of Dr. Wilson anticipates and includes the "unity of the body," so much insisted on by Mr. Macilwain. We apprehend, indeed, that Mr. M. had overloooked the work of Dr. Wilson, and probably did not advert with attention to the able paper of Dr. Alison, reviewed in our Journal of Jan. 1827. *Eds.* 1836.

currence, as to be noticed as things of course ; as, for instance, the gastro-cerebral, the cutaneo-gastric, and the utero-gastric sympathies.

These morbid sympathetic impressions on distant organs or parts are sometimes attended with pain, and sometimes not. In the *latter* case, the original irritation only increases or decreases the excitement in the sympathising structure. Thus we see the *vascular* system sometimes thrown into *inordinate* action by acrimonious irritation in the alimentary canal; at other times, the action of the heart is almost suspended by the application of offensive substances to the nerves of the stomach. But when the sympathetic irritation arises to the degree of *spasm*, which, in fact, is only the highest degree of contractile action, in a muscular or fibrous structure, then we have *pain.*

"Wherever, therefore, muscular fibres exist, morbid spasm may also exist; consequently, not only the fibres of large muscles, but the smallest fibrous threads of all the membranous parts, are also susceptible of spasm from irritation, applied either directly or indirectly from morbid sympathy.

It is further to be observed, that although the most violent spasms of large muscles are seldom, if ever, succeeded by local inflammation, as is instanced in epilepsy, hysteria, &c.; yet, when the muscular texture of a membrane becomes the seat of a sympathetic spasm, *local inflammation of that membrane is speedily induced, the coats of its capillary vessels being probably also soon included in the sympathetic influence.*" 22.

This is an important doctrine, which is ever to be borne in mind by those who separate spasm from inflammation, and never dream of the *latter* when they have to combat the former.

With regard to the vinculum, as Dr. W. justly observes, connecting the sympathising part with that where the irritation is applied, we remain totally in the dark; and, therefore, all we have in our power on this point is, by careful observation, to mark demonstrable facts; and these, by candid induction, ought to lead to correct practice.

The extensive influence which a healthy or diseased state of the digestive organs, and of the stomach in particular, exercises over the whole human frame, mental and corporeal, has long been, and still continues to be, the subject of observation and wonder among pathologists. When, indeed, we consider that the mucous membrane, stretching from the mouth to the anus, and lining so many important hollow viscera, is a secreting surface, studded with innumerable nervous or sentient papillæ, and exposed daily to a mass of heterogeneous and stimulating matters, as well what is poured in, in the shape of food and drink, as poured forth from the mucous glands themselves, in the shape of morbid secretion, it is not to be wondered at that the primæ viæ are the seat of most of those primary irritations which are productive of disease. The morbid sympathies which subsist between the stomach and various distant parts are doubtless reciprocal; but those which radiate *from* the gastric centre, would appear to be more powerful than those which are propagated from distant parts to it. It must also be remembered, as was hinted before, that irritation in the fibrous structures of the membranes lining the hollow viscera, and entering into the composition of joints, &c. will induce first spasmodic action; and whenever *this* arrives at a certain height, inflammation in the structures under consideration. This local inflammatory movement will soon draw the heart and vascular system into increased action, by morbid sympathy—and thus a febrile state is induced. The following passage from Dr. Wilson depicts precisely our own sentiments, and accords with our own practice and experience.

" In fact, the operation of the *laws of sympathy*, through the nervous influence on the living system, is a thing demonstrable to the senses; and any theories which I have ventured to offer on their pathology, are very nearly as much so to an unbiassed mind. At least, this I will assert for myself, that, to my conception, they approach far nearer to the certainty of demonstration, than any other pathology I have met with on the same subjects, and have served to *direct my own practice*, in a satisfactory manner, through a mist of insulated facts and general assertions ; while every additional year's experience has served more and more to strengthen my opinions." 36.

We believe that Darwin, the great father of the sympathetic doctrines of modern times, was the most *successful* practitioner that England or the world ever produced. There was scarcely a town on the Continent of Europe from whence he was not consulted, either personally or by letter ; and we have it from good authority, that his success was mainly owing to the *multiplied resources* which the sympathetic views of disease opened out to him. In our own practice, for many years past, we have had good reasons for pursuing the doctrines and practices of Darwin ; and we seriously recommend them to the rising generation, who are not yet so blinded by prejudice or pride, as to be beyond the reception of any thing in the shape of advice from others.

In the fourth Letter, our author enters on the consideration of febrile diseases. He considers that " a permanently-increased action of the heart and arteries" is a *sine qua non* in fever, no other phenomenon being invariably present. We think, however, that a *deranged action* of the heart and arteries is a more permanent symptom in fever, than an *increased* action ; but we shall not cavil about definitions. It is of more consequence to bear in mind, that this increased (deranged) action of the heart and arteries may arise

" From irritation, applied to the nerves of some distant part of the body, between which and the vascular system a primary affinity subsists, exciting that system into violent action by morbid sympathy; or from the effects of something of an irritating nature having been received into, and circulating with the blood, and thereby directly applied to the internal surface of the heart and bloodvessels themselves. But the establishment of a febrile state of the system, as derived from remote causes within the body, appears very demonstrably to acknowledge the following primary distinctions :

1st. As derived from the effects of acrid matter applied to some department of the nervous system, and exciting a morbid action of the heart and arteries by morbid sympathy ; but such a remote cause, experience shows, is most commonly seated in the digestive organs.

2d. As derived from the effects of irritation, arising from organic obstruction, somewhere existing in the body, and exciting inc1eased action of the heart and arteries, by morbid sympathy.

3d. As derived from the effects of something of an irritating nature received into, and circulated with the blood.

4th. As derived from passions of the mind, exciting an increased action of the vascular system, by morbid sympathy.

5th. As derived from two or more of these primary causes of fever acting at the same time." 43.

Our author candidly acknowledges that we are totally ignorant why gastric irritation should in one person excite the vascular system, and in another the voluntary muscular system; but we are certain that such is the fact. Dr. W. then proceeds to the consideration of gastric fever, as the most common, and, at the same time, the most simple form of fever. He considers the irritation in the stomach as propagated, by morbid sympathy, to the heart and arteries. In this species of fever, the first signs of any departure from health are of that kind which point out impaired action and morbid irritation in the digestive organs, as is evinced by the precursory phenomena of diminished appetite—weight at the præcordia—abdominal distention—thirst—parched mouth, and furred tongue. To these are commonly added, signs of impaired and irregular action in the nervous system, arising from morbid sympathies with the primæ viæ, such as languor, disturbed sleep, headache, dry skin, flushings and chills. This is the prevailing fever of infantile years; and, if taken in time, may soon be checked, merely by the ejection of the source of irritation from the primæ viæ; another proof of the truth of the doctrine in question.

The two great indications, then, in gastric fever are, the evacuation of the morbid secretions from the line of the alimentary canal, and the reduction of morbid temperature on the surface. Dr. W. here takes occasion to object to the unreasonable prejudice which has lately existed against emetics in this country, chiefly engendered, we conceive, by their deleterious effects in the hotter regions of the earth, where the gastric and biliary systems are ordinarily in a state of irritability, which is greatly exasperated in the fevers of those climates, and of course where emetics are improper. But in this country it is very different, and physicians lose a powerful febrifuge by the disuse of emetic tartar in particular.

"Nor are antimonial emetics attended by any hazard, nor even by more severe nausea than ipecacuan, unless it happens by an over-dose, or by mismanagement during its operation. One grain of ant. tart. for an adult, will generally nauseate the stomach in half an hour; when, without waiting longer, the stomach should be distended by drinking warm water, and vomiting is promoted by irritating the fauces a little with a feather. One half-glass of wine after every fit of vomiting will assist its operation." "It is by a prudent and free administration, then, of antimonial emetics, along with mercurial laxatives, assisted by the neutral salts, that the first and great indication of cure is here to be accomplished." 60.

The Doctor takes this opportunity of expressing his opinion, that the *debility* in these fevers is only apparent, not real; and of course to be removed or prevented by evacuations, which take off the load from the spring of life. He justly remarks, that mere *aperients* will not do; but that "very free evacuations are always necessary, procured by the help of the most active *purgatives*, administered in appropriate doses; remarking the nature of what comes off, till it puts on a healthy appearance, a circumstance which usually attends a solution of the fever, with its accompanying train of symptoms."

Our author next turns to the consideration of fever, as dependent on "idiopathic organic obstruction;" viz. topical inflammations, from whatever occasional source they may be derived, as local stimuli, wounds, bruises, &c.

"The application of excessive cold, or of burning heat, &c. forming obstructions in the capillary vessels of the vascular system, and exhibiting what is designed local inflammation; from whence, by a sympathetic affinity, the heart and arteries are drawn into violent action, and it thereby becomes a primary cause of fever." 71.

After some ingenious remarks on the nature of the topical inflammation itself, in which he concludes that the obstruction to the free stream of blood is seated in the capillary extremities of the veins, our author observes, that, with respect to the *treatment* of fever in these cases,—

"Large and general bleedings, as also topical bleeding, whenever it can be applied, are perhaps the most powerful means; and along with these, aperient medicines. But, whenever an acting morbid cause is observed to exist in the digestive organs, a free discharge from these, *by the most active evacuants*, is a measure of great importance, from the mutual sympathy betwixt the stomach and every part of the body. As to external applications, I have always observed tepid fomentations to be the most useful, and the most soothing to the patient's feelings." 80.

The ratio symptomatum, and pathology of typhus are next descanted on by our author, with much ingenuity and acuteness; and, although we cannot agree with him on all points here, particularly in his opinion that the gastric and biliary secretions are greatly *increased* at the beginning of typhus, yet his doctrines all tend to support the propriety of the depletory or anti-stimulant plan of treatment now superseding the Brunonian mania. Dr. W. believes, and we unite our suffrage to his, that cinchona stops the paroxysms of an ague, not through the medium of the circulation, but by its tonic, and perhaps sedative effects on the nerves of the stomach. Indeed, it is only in this way that the salutary operation of several other remedies in intermittent fever can be accounted for.

In respect to our author's *methodus medendi*, we think it is not quite energetic enough; though, probably, in the part where he resides, and where fevers are not likely to assume the graver types, the practice may be good enough.

Dr. Wilson's long chain of reasoning on influenza we must pass over, in order to pay some attention to his opinions on acute rheumatism. Here the sceptics of the day will be ready to say, that Dr. W. far outstrips our illustrious London pathologist, Mr. Abernethy, in attributing a variety of diseases to derangement of the *digestive organs*. Although we are not disposed to go the lengths of either Dr. W. or Mr. Abernethy, on this subject, yet we are convinced that there is much truth and justice in the doctrines of both these gentlemen.

"Acute rheumatism (says Dr. W.) in its progress, appears to include two stages of diseases; the first of which is *gastric fever*, attended by a painful state of the membranes surrounding certain joints, excited by a spasmodic affection fixed on them, in consequence of morbid sympathy, with irritation in the primæ viæ. The second stage is formed by an inflamed state of these membranes, created by the continued action of the same sympathetie affinity, which inflammation is occasionally so considerable as to produce effusions in the cellular membrane, and even the bones of the articulation become sometimes inflamed, eventually giving rise to anchylosis, and other diseases of the joints." 221.

On these principles, Dr. Wilson employs liberal evacuations from the primæ viæ, especially by calomel and antimony, aided occasionally by the neutral salts, particularly the tartrite of potass, or phosphate of soda, "till the signs of a loaded state of the digestive organs shall disappear, or at least shall have greatly abated. Anodynes, which have the effect of suspending spasm, and of procuring a state of rest, are useful from the beginning; but their usefulness is most eminent after the primary irritation is lessened."*

Our author employs bark and steel, when the febrile state is subsided. For the local inflammation, he trusts principally to topical bleedings and fomentations, "a little more than blood warm." He also recommends warm anodyne poultices, composed of the common forms, with opium or cicuta added. He is an advocate for calomel and opium in acute rheumatism.

From this disease, the transition to gout is easy. Our author not only supports the idea of a strong affinity between gout and rheumatism, but comes to the conclusion that both diseases mainly depend on derangement in the digestive organs.

"In contemplating the above premises, it is obvious that there appears no other demonstrable cause of vascular excitement, but that which, by unequivocal signs, is shewn to exist in the digestive organs; therefore, this unavoidable conclusion is likewise obvious, that the febrile paroxysm excited in the attack of a fit of gout must be viewed as real gastric fever; and farther, that any other suppositious principle, as inherent in the system, but which is undemonstrable as a primary agent, or remote cause of the disease, may fairly be esteemed as imaginary."

This doctrine, indeed, is very little different from that inculcated in some of the latest and best works on gout. In respect to the treatment, Dr. W. justly observes, that over the predisposition or idiosyncrasy of the patient, medicine has little control; yet many of the inordinate movements in gout may be soothed and moderated—"especially by evacuations from the stomach and bowels."—Dr. W. considers, also, that such medicines as act upon the skin are extremely beneficial; as the liq. ant. tart. and tinct. opii, accompanied with diluting drinks. The acetate of ammonia, also, given in tepid drinks, is useful for the same purpose. In respect to local applications, "the tepid warmth of an emollient poultice, void of every kind of acrimony, is the most suitable; or, tepid emollient fomentations, covering the parts afterwards with warm flannel. To these may be added, topical bleeding with leeches."

In the intervals of the paroxysms, Dr. W. recommends the greatest attention to the state of the stomach and bowels, particularly by laxative medicines.

"Of the last, calomel is the most efficient, and ought to form the chief basis of the measures taken for clearing the lower intestines, accompanied with the occasional use of some saline purge."

Emetics, he thinks, are valuable remedies in the remissions of the disease. He condemns the external application of cold, lest "some other organs, which may be next in sympathetic affinity with the seat of irritation, become affected with its influence; and if this shall happen to be any vital organ, the result may be fatal."

---

* Dr. W. was probably unacquainted with the virtues of colchicum at the time he wrote.—*Eds.* 1836.

Dr. W. next proceeds to draw a parallel between gout and erysipelas—a parallel which has been very fully drawn by the continental physicians.

"A gentleman has had an ulcer on his leg for many years. Along with the ulcerated leg, he is, at the same time, subject to bilious accumulations in the primæ viæ; but these accumulations seldom occur without producing a febrile paroxysm, attended by extensive erysipelas around the ulcer; under the influence of which he has occasionally laboured for two weeks at a time, but without any medical aid, further than what he derived from some gentle aperient medicine to preserve his bowels soft. At last, when under one of these feverish fits, he was persuaded to take, though reluctantly, an antimonial emetic, and even to repeat it, followed with a mercurial laxative; by which means he threw off a great quantity of offensive bilious fluid, after which the inflammation and fever very quickly abated. Since this time, as the ulcer on his leg still remains, and continuing still subject to bilious complaints, he has become aware of the approaching illness; and being now convinced of the benefit attending this practice, he has, by seasonable evacuation, saved himself from many a fit of fever and erysipelas." 376.

Dr. W. observes that irritating matters, and consequently irritation, cannot be expected to be removed from the long line of the digestive organs by a few evacuations, whether of the emetic or cathartic class. Part of the offending matter will often lurk in the duodenum, or some of the sinuosities of the other intestines, while the ordinary discharges by the rectum are suffered to pass along. Additional morbid secretions may also be collected anew—

"Therefore the evacuations ought to be persevered in till the signs of irritation in the stomach shall disappear, and till the discharges from the rectum shall assume a healthy aspect; otherwise, the portion of vitiated matter which is retained, will assuredly tend to prolong the disease, and disappoint expectations." 379.

*Angina Pectoris.* Dr. Wilson thinks that post-mortem researches "have offered no certain information, nor discovered any organic disease within the thorax, which might be referred to as the direct cause of its symptoms." Here we cannot coincide with our author. The Histories and Dissections of Dr. Parry, and cases which have since been published, afford very satisfactory evidence that all the symptoms of angina pectoris have *sometimes* resulted from organic disease. We are ready to admit, however, that all the phenomena of syncope anginosa have *also* occurred, and terminated even in death, without any disease of structure being cognizable to the senses. This must lead unbiassed minds to the conclusion, that it is sometimes a disease of structure, giving rise to periodical lesion of function—and, at others, only a derangement of *function* in the respiratory and circulating organs, leaving no perceptible trace of its previous existence after the thread of life is snapped. In the latter cases, we can only suppose it to be of a spasmodic nature, resulting from arthritic or rheumatic irritation misplaced, and not yet amounting to ostensible organic change in any tissue or vessel. It is in this light that our author views it, and we shall present his ideas on the subject.

"In the course of my own experience, I have seen but a few cases of this kind; but from my observations on these few, I have uniformly discovered, that at the commencement of the disease, the digestive organs were in a loaded state; as also, from farther inquiry that they had previously been so. But from considering the strong disposition to sympathetic action which subsists, in some individuals, betwixt these organs and the heart, as well as other organs within the thorax, together with a general review of the predominant symptoms, I cannot avoid drawing this inference, that angina pectoris is a disease which also arises from a morbid sympathy, with a primary cause seated in the primæ viæ." 385.

With respect to the particular seat of the spasm, Dr. W. thinks, that it is doubtless either in the heart itself, or the adjoining great bloodvessels, or perhaps in both at the same time; he also thinks that, from the fixed severe pain under the sternum, a little to the left side, and, at times, quite across to the back, the mediastinum may be very materially included in the spasmodic affection, perhaps in a primary manner, as in arthritic or rheumatic translations. It is certain, indeed, that angina pectoris chiefly attacks those of a gouty or rheumatic character. We shall introduce an interesting case in elucidation.

"April 18th, 1777. Mrs. ———, aged 50 years, complains of an intolerably severe stricture extending quite across her breast, attended by a severe pain under the middle of the sternum, which communicates itself to both her arms in a violent manner, but confined to a spot about the middle of the humerus; accompanied with a feeling of suffocation, and a most irregular, soft, intermitting pulse. In this state she continues for the space of ten or fifteen minutes, when the symptoms gradually abate, and she feels free from any complaint, excepting weakness, her spirits being much exhausted. Her appetite for food is impaired; her tongue much furred, with thirst; bad taste in her mouth; weight at the præcordia, and restless nights; her pulse, excepting when under the fit, being natural. She is of a spare habit, and subject to atonic rheumatism in her arms and limbs, and has been repeatedly affected with violent periodical headaches; she has been complaining in this way for some days. At first, the fits returned twice or thrice in the twenty-four hours, with unequal violence; but she feels them increasing in frequency as well as in violence, and now a recumbent posture is certain of producing them at any time.

She had, on some former occasion, been alarmed by the severe operation of an emetic, and I found, at present, that her aversion to it could not, as yet, be conquered. I was on that account left to the effect of laxatives alone, for the purpose of clearing out her stomach and bowels. These were accompanied with opiates, and a blister on her breast. The first cathartic dose produced some bilious stools; and a repetition of it every morning, with a powerful opiate three times daily, had the effect of mitigating the violence of the disease for a few days; after which it returned with increased violence, both in length and frequency of the fits, accompanied with faintness; her pulse was extremely confused, and she described her feelings of anguish as intolerable. She had now been out of bed for eight nights and days, because any attempt at a reclining posture was certain of producing a paroxysm; on which account she was compelled to spend the whole of her time in her chair, and notwithstanding the opiates, she slept very little. I now at last prevailed on her to use an emetic of ant. tart. and she discharged a great quantity of acrid, bilious-coloured fluid. On the succeeding night she enjoyed more sleep, but still could not bear a lying posture. On the 27th the fits less severe, and less frequent; she continues the opiates, and, on the 28th, took a laxative dose. On the 29th relapsed, and took another emetic; threw up much bile, and was again relieved.

May 2d, took another emetic yesterday, and has had no fit for twenty-four hours—can sleep in bed. May 4th, has had some slight returns yesterday, and repeated the emetic; discharged more offensive bilious fluid, and is better. May 10th, continues better; her tongue is clean, and other signs of a loaded stomach, &c. gone off. May 29th, has taken stomachic bitters, with the daily use of aperient pills; and by the help of moderate exercise, and restorative diet, remains quite well. She had afterwards repeated slighter attacks of the same complaints, which were always removed by a discharge of offensive matter from the intestinal canal, procured by the same means, and died at last of another disease in the year 1790." 390.

Finally, our author is convinced, from a careful attention to the phenomena of croup, that the primary seat of irritation is in the digestive organs, whence is generated, by morbid sympathy, a spasmodic affection of the glottis, ending in inflammation of the mucous membrane of the larynx and trachea, ultimately destroying the action of the lungs, by rendering them impervious. Acting from these premises, Dr. Wilson asserts, that our great indication is to clear the stomach and alimentary canal, as early as possible, by emetics and mercurial cathartics.

Indeed, I believe these medicines to be the most efficient means hitherto known of speedily and effectually accomplishing, on any occasion, this important object." 409.

This plan will often, he thinks, check the disease in its nascent movements, and before actual inflammation has commenced; but we believe that such nascent movements, or, in other words, the *spasmodic* stage, is rarely witnessed by medical men: and, consequently, it is against the inflammation and exudation from the surface of the mucous membrane that we have principally to guard. Hence, while we take every opportunity of acting on the alimentary canal by emetics and calomel, we must detract blood from the throat, and counter-irritate in the neighbourhood, with all possible expedition and energy.

We have now presented a full analysis of Dr. Wilson's work. Its greatest fault, or, more correctly speaking, its misfortune, is the diffuseness of the style. In one fourth part of the space, he might have given the same matter in an infinitely stronger point of view. Indeed, *medical economy* in medical literature is too much neglected. We are fully convinced, that the man who endeavours to diffuse professional knowledge on the easiest terms will, in the end, be the greatest benefactor to medical society. The higher classes have not time to peruse, and the inferior orders have not money to purchase, voluminous publications; and hence the stream of science flows in narrow and extremely confined channels. We could illustrate this fact by anecdotes, which would be scarcely credited by the well-informed members of the profession, but which are strictly true. We were lately in conversation with one of the first-rate medical practitioners in the greatest city of the world, who neither knew the name nor any part of the writings of one of the greatest ornaments of medical science at the present period! It would be equally mortifying and discouraging to the really zealous cultivators of medical science, if they knew the narrow limits in which their labours move. It is true that medical works take a large *circuit*, in consequence of medical clubs and societies; but the *actual readers* are few indeed!

This, in some degree, is owing to the expansion of little matter into large space, and to the consequent expence of purchase; so that, comparatively speaking, medical writings are little known, excepting through the medium of Journals.

